# Smart bioadhesives for wound healing and closure

**DOI:** 10.1016/j.bioactmat.2022.04.020

**Published:** 2022-04-26

**Authors:** Jia Zhu, Honglei Zhou, Ethan Michael Gerhard, Senhao Zhang, Flor Itzel Parra Rodríguez, Taisong Pan, Hongbo Yang, Yuan Lin, Jian Yang, Huanyu Cheng

**Affiliations:** a Department of Engineering Science and Mechanics, The Pennsylvania State University, University Park, PA, 16802, USA; b AML, Department of Engineering Mechanics, Tsinghua University, Beijing, 100084, China; c Institute of Flexible Electronics Technology of THU, Zhejiang, Jiaxing, 314000, China; d Department of Biomedical Engineering, The Pennsylvania State University, University Park, PA, 16802, USA; e Suzhou Institute of Biomedical Engineering and Technology, Chinese Academy of Science, Suzhou, 215011, PR China; f School of Materials and Energy, State Key Laboratory of Electronic Thin Films and Integrated Devices, University of Electronic Science and Technology of China, Chengdu, 610054, PR China; g Materials Research Institute, The Pennsylvania State University, University Park, PA, 16802, USA

**Keywords:** Smart bioadhesives, Immunomodulatory bioadhesives, Mechanically/electrically active bioadhesives, Closed-loop system, On-demand treatments, Wound healing and closure

## Abstract

The high demand for rapid wound healing has spurred the development of multifunctional and smart bioadhesives with strong bioadhesion, antibacterial effect, real-time sensing, wireless communication, and on-demand treatment capabilities. Bioadhesives with bio-inspired structures and chemicals have shown unprecedented adhesion strengths, as well as tunable optical, electrical, and bio-dissolvable properties. Accelerated wound healing has been achieved via directly released antibacterial and growth factors, material or drug-induced host immune responses, and delivery of curative cells. Most recently, the integration of biosensing and treatment modules with wireless units in a closed-loop system yielded smart bioadhesives, allowing real-time sensing of the physiological conditions (e.g., pH, temperature, uric acid, glucose, and cytokine) with iterative feedback for drastically enhanced, stage-specific wound healing by triggering drug delivery and treatment to avoid infection or prolonged inflammation. Despite rapid advances in the burgeoning field, challenges still exist in the design and fabrication of integrated systems, particularly for chronic wounds, presenting significant opportunities for the future development of next-generation smart materials and systems.

## Highlights

• Rational material engineering of bioadhesives with optimized mechanical and curative properties.
• Incorporation of biosensing allows real-time and precise evaluation of the healing stage.
• Closed-loop, smart bioadhesives that integrate wireless sensing and treatment hold great potential for chronic wound healing.

## Graphical abstract

**Figure:**
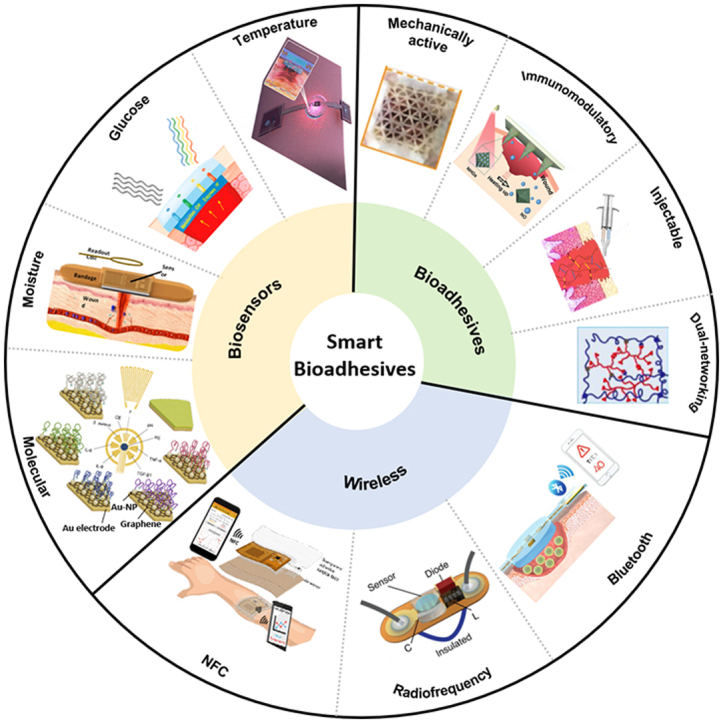

## 1 Introduction

Normal wound healing is a complex physiological process consisting of four well-organized stages: hemostasis, inflammation, proliferation, and remodeling, with the involvement of different cells, including platelets, inflammatory (e.g., neutrophils and monocytes), immune, endothelial, and circulating progenitor cells [1]. During the initial hemostasis phase, platelets are activated to form a fibrin clot through pro-coagulants and the release of prothrombin to cease blood flow [1]. Next, blood vessels dilate during the inflammatory phase, allowing the leakage of essential cells, antibodies, growth factors, enzymes, and nutrients into the wound area [[2], [3], [4]]. As the inflammation phase ends, endothelial cells and blood vessels start to proliferate. New blood vessels transport oxygen and nutrients to promote fibroblast proliferation, while myofibroblasts differentiated from fibroblasts deposit extracellular matrix through interactions with their microenvironment and mechanical signaling. Collagen is remodeled from type III to type I during the remodeling stage, followed by alignment along the tension lines, to restore tensile strength from the reorganization and degradation of cells previously used to repair the wound, transitioning from reparative to functional structures/tissues. The improper interaction with their microenvironment leads to the persistence of myofibroblasts and consequently scar formation. However, chronic wounds (e.g., venous, diabetic, and pressure ulcers) are often stalled in the inflammation stage due to impaired cellular function, diminished growth factor populations, severe infection, or autoimmune failure. Motivated by the limited innate healing capability of chronic wounds and the inefficiency of repetitious topical drug administration, wound dressings have increasingly been utilized, ranging from early drug impregnated non-degradable materials such as gauze, polyurethane foams to bioadhesives.

Beginning with natural adhesives (whose main function is sealing), the exploration of bioadhesives has extended to bio-inspired and synthetic materials in the pursuit of optimized mechanical, biocompatible, and curative properties. The primary requirement for bioadhesives is strong adhesion to human tissues and sufficient cohesion to sustain shear deformations [5,6]. However, these two properties in traditional materials are often in conflict with each other. By modifying side groups for covalent or non-covalent interactions, controlling the crosslinking density, or introducing dual networks (DNs), synthetic bioadhesives can provide tunable mechanical properties and exhibit an improved balance between adhesion and cohesion [7]. The usefulness of introducing DNs has been evidenced by the tunability of bioadhesives in biodegradability, drug loading capabilities, and drug release profiles. For example, hydrophilic bioadhesives are conventionally thought to be only suitable for hydrophilic drug loading and delivery; however, delivery of hydrophobic drugs can be enabled via incorporation of hydrophobic regions into the polymer backbone via chemical conjugation or host-guest interactions with bound cyclodextrin moieties [[8], [9], [10]]. Curative hydrogel bioadhesives loaded with immunomodulatory factors can regulate the secretion of various signaling molecules (e.g., growth factors and cytokines), trigger specific immune responses, and enable stage-specific healing mechanisms [4,11,12]. Hydrogel bioadhesives in the injection form with post-polymerization capabilities extend their applications from skins to inner tissues or organs that have limited accessibility [[13], [14], [15]]. Besides these chemical and biomedical curative effects, physical therapies (e.g., mechanical, electrical, optical, and thermal stimuli) have demonstrated great potential in accelerating wound healing.

**Fig. 1:**
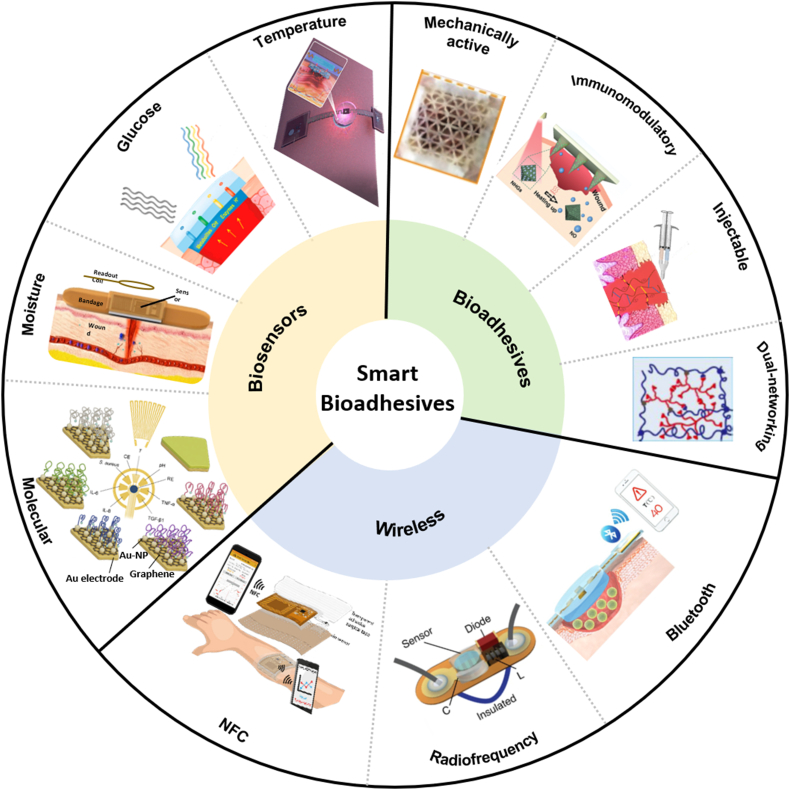
Smart bioadhesives with biosensing, drug delivery, and wireless communication modules for a closed-loop system. Representative state-of-art bioadhesives include dual-network hydrogels, injectable hydrogels, immunomodulatory hydrogels, and mechanically active adhesives. Various physiological sensors, including temperature, glucose, moisture, and immune indicator sensors, enable real-time and precise evaluation of wound healing status. The incorporation of wireless technology, such as near field communication (NFC), radiofrequency, and Bluetooth, enables wireless sensing and remote manipulation. Reprinted with permission from Refs. [17,18,20,25,26,48,73,86,121,137,138].

Meanwhile, it is vital to monitor physiological conditions (e.g., temperature and moisture) [16,17] and relevant biomarkers (e.g., pH, glucose, uric, oxygen, nitric oxide, and cytokines) [[18], [19], [20]] to provide a real-time and precise evaluation of wound healing processes. Previous evaluation of wound healing stages based on visual observation and clinician's experience in these cases is incapable of providing real-time and precise information, which may lead to delayed treatments and unexpected outcomes. Integrated sensing eliminates the need for replacement and removal of dressings for inspection, improving evaluation precision and reducing pain, especially for those with chronic wounds. Further, wireless transmission by near field communication (NFC), Wi-Fi, Bluetooth, and Cloud can remove wire connections and improve portability and practicability [[21], [22], [23], [24], [25]]. Radiofrequency (RF) antennas can also be leveraged for long-range sensing [26]. Collected sensing data can then be analyzed to trigger treatments on demand. Multifunctional bioadhesives are non-invasive, easy to deploy, allow real-time and precise evaluations, and are capable of carrying drugs for on-demand delivery or applying physical therapies (Fig. 1). In this review, we will first introduce recently developed multifunctional hydrogel bioadhesives and mechanically or/and electrically active bioadhesives. Next, we will discuss the important role of biosensors and bioelectronics in evaluating the wound healing processes in real-time. Finally, methods for the integration of sensing and treatment with wireless communication units in a closed-loop manner toward enabling new classes of smart bioadhesives will be introduced.

## 2 Working principles of bioadhesives

Coined in 1970, the term bioadhesion describes the adhesion of natural (including those derived from living organisms) or synthetic materials to biological tissues. An effective bioadhesive needs to: 1) adhere to biological tissues and, 2) hold together the two sides of a wound against motion-induced shear forces [6]. The former originates from interactions at the bioadhesive/tissue interface and the latter relies on crosslinking mediated bioadhesive cohesion. Interfacial adhesion can result from mechanical interlocking, chain entanglement (or diffusion), electrostatic force, or intermolecular bonding (absorption and wetting) [4,27,28]. Mechanical interlocking [29] or chain entanglement [30] can take place at the interface when bioadhesives diffuse onto the irregular surface or infiltrate inside the tissue due to the concentration difference. Electrostatic force originates from the electric double layer that is formed due to different electron affinities of bioadhesives and tissues. Intermolecular bonding at the interface includes major bonds (e.g., metallic, ionic, and covalent) and secondary bonds (e.g., van der Waals interactions and hydrogen bonds), which are related to wetting and absorption at the interface. Though secondary bonds widely exist, major bonds often have higher bonding energy and thus larger adhesion strengths. Because increasing the effective contact area can lead to improved adhesion, it is highly desirable to explore bioadhesives with good wettability on biological tissues for increased effective contact area [5]. Wetting of bioadhesives on tissues is mainly driven by the interfacial surface energy at the bioadhesive/tissue interface. The fundamental importance of interfacial interaction in adhesion suggests the high potential of bottom-up polymer engineering of the main network and incorporation of functional side groups to optimize the adhesive strength.

Cohesion refers to the ability of the bioadhesive to maintain its shape under shear forces, originating from crosslinking and interlocking effects during polymerization. Considering the relatively large shear deformation of human tissues in the dynamic physiological environment, sufficient cohesion is critical. Higher crosslinking strength and density result in larger cohesion and stiffness, but also reduce conformal ability, wettability, and penetration ability, leading to reduced adhesion. On the other hand, polymers with low molecular weight have excellent wettability, diffusion, and absorption capabilities, but low cohesion. The conflict between adhesion and cohesion is widely observed in natural adhesives. Therefore, rational material design in synthetic bioadhesives is often needed to achieve balanced adhesion and cohesion for different targeted applications, such as using different functional groups for cohesion and adhesion or introducing DNs [7,27,30]. Typically, Young's moduli of bio-tissues are in the range of several kPa to MPa [31,32]. The mechanical stiffness of bioadhesives should match the bio-tissue to avoid discomfort. It is also necessary to consider the local strain state of bio-tissues induced by body movements.

Early natural bioadhesives include fibrin, starch, collagen, chitosan [33], fibroin [34], cellulose, and various gums (e.g., guar, pectin, and alginate). Despite intrinsic biocompatibility, the fixed chemical structure of natural adhesives (e.g., the molecular weight of monomers and crosslinking level) doesn't provide tunable mechanical properties, resulting in unsuitable stiffness [35] or poor adhesion [36]. As a parallel direction to chemical-induced adhesion, biomimetic structures inspired by natural creatures have recently been an exciting area, demonstrating controllable or even reversible adhesion via dedicated microstructures. Typical microstructures include gecko feet, beetle footpads, octopus suction cups [37,38], slug footpads, and endoparasite proboscis [39]. Temporary and dry adhesion in these creatures is based on van der Waals forces due to a huge collective surface area of contact [40], such as the periodic-arranged foot-hairs in gecko feet, mushroom-shaped structures in beetle footpads, or the negative pressure in microcavities, such as octopus suction cups. Of note, such structurally engineered patches also show adhesion to wet faces due to capillary force and negative pressure [38,41].

In order to better engineer bioadhesives, the underlying adhesion mechanism has to be explored. The role and effectiveness of varying terminal groups (e.g., OH-, COOH-, NH_2_-, and CH_3_-) in bioadhesives to cell adhesion have been systematically studied using self-assembly layers with different terminations [42]. The OH- group is found to have the highest adhesion strength to cells, followed by COOH-, NH_2_-, and then CH_3_-. Compared with the hydrophobic CH_3_-, hydrophilic groups (e.g., OH-, COOH-, and NH_2_-) have increased flowability, wettability, and penetration through hydrogen bonding [4,6]. These principles have spurred the design and preparation of synthetic bioadhesives such as cyanoacrylate and its variants (e.g., methyl 2-cyanoacrylate, ethyl 2-cyanoacrylate, n-butyl cyanoacrylate, octyl cyanoacrylate, and 2-octyl cyanoacrylate) [43]. Since FDA approval in 1998, 2-octyl cyanoacrylate (Dermabond; Ethicon) has been widely used for closing topical skin incisions [44]. Despite strong adhesion and high water resistance, cyanoacrylate-based bioadhesives are too brittle to be used for joint wounds and their potential clinical use for internal wounds in terms of biocompatibility still needs to be carefully examined [45]. Hydrogels have similar structures and mechanical properties to human tissues due to their high content of water and can accelerate wound healing by maintaining a moist environment [46]. They can be easily loaded with hydrophilic drugs and effectively release upon thermal stimuli for treatments [47]. Due to their great tunability, hydrogel bioadhesives have been functionalized or customized through material and structural engineering, such as dual-networking hydrogels, immunomodulatory hydrogels, and injectable hydrogels, for specific application scenarios. A summary of state-of-art strategies for wound healing and their properties was given in Table 1.

**Table 1:** Different strategies for wound healing and their properties.

Category	Examples	Advantages	Disadvantages	Reference
Traditional dressing	gauze dressing, film dressing, foam dressing	commercialized, mature	need additional adhesion or fixture and replacement, not suitable for internal wounds	[139]
Natural bioadhesive	fibrin, starch, collagen, chitosan, fibroin, cellulose, alginate	biocompatibility, easy to obtain	insufficient adhesion or cohesion strength, lack of curative effects	[33,34]
Structurally engineered bioadhesive	gecko feet, beetle footpads, octopus suction cups	reversible adhesion and release	high fabrication cost, low adhesion to wet surfaces	[41,140]
Synthetic bioadhesive	cyanoacrylate	strong adhesion, rapid cure	dry adhesion, low biocompatibility, brittle	[44]
	hydrogels	great tunability, high water content, drug-loading capability	need rational designs	[4,10]

## 3 Hydrogel bioadhesives

### 3.1 Dual-networking hydrogels

**Fig. 2:**
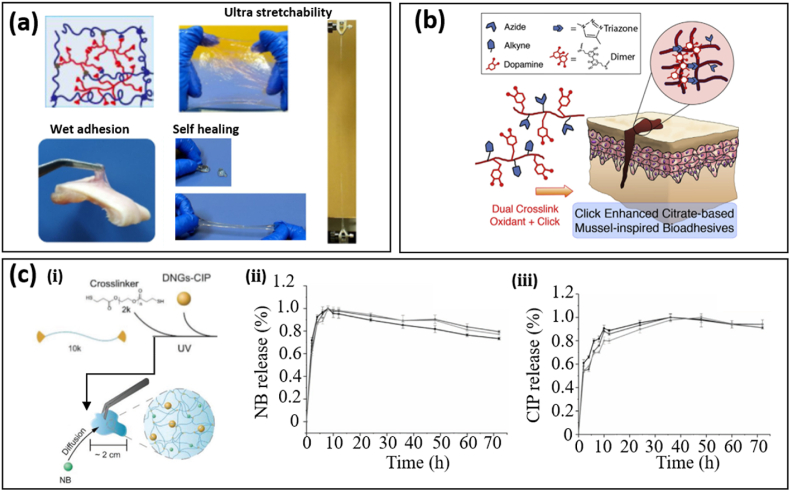
Hydrogel bioadhesives with dual-networks (DNs) for optimized mechanical properties or drug loading capabilities. (a) An ultra stretchable, self-healing, and UV-curable hydrogel bioadhesive based on PAM and PEI DNs. The reversible hydrogen (breaking and recombining) bond contributes to ultra-stretchability and self-healing. Reprinted with permission from Ref. [48]. **(b)** Click chemistry was introduced to improve the cohesion and adhesion of DOPA-based bioadhesives in a wet environment. The additional crosslinking due to the formation of triazine rings between azide- and alkyne-functionalized DOPA-based bioadhesives serves as cohesive strength-improving moieties and spares more catechol groups in dopamine for adhesion. Reprinted with permission from Ref. [59]. **(c) (i)** Dual-drug delivery (hydrophobic ciprofloxacin (CIP) and hydrophilic novobiocin sodium salt (NB)) achieved by introducing dendritic nanogels (DNGs) with hydrophobic cores into a hydrophilic polyethylene glycol (PEG) network. The accumulative release rate of CIP **(ⅱ)** and NB **(ⅲ)** with time. Reprinted with permission from Ref. [63].

The concept of hydrogels is very broad, covering the aforementioned natural derivatives (such as chitosan, alginate, and collagen) and synthetic polymers. Despite low adhesion and strength in early hydrogels, these limitations have been eliminated in synthetic hydrogels by using new monomers, modifying functional groups along the backbone [14], and/or introducing DNs [48]. Of note, DN design is the most effective and widely adopted method in optimizing the mechanical, optical, electrical, and drug loading/releasing properties of hydrogel bioadhesives. In one representative example, the good mechanical properties of hydrogels, including stretchability and adhesion, have been demonstrated in a DN based on acrylamide (AM) monomer and hyper-branched polyethylenimine (PEI) polymer. It exhibits high stretchability (over 5500%), strong adhesion strength to pigskin (7 kPa), and fast self-healing (less than 30 s) [48] (Fig. 2a). The ultra-stretchability and self-healing originate from dynamic hydrogen bonding between the polyacrylamide (PAM) and PEI network. Increasing the PEI content in PEI/PAM hydrogels leads to high stretchability at the expense of mechanical stiffness. Reversible hydrogen bonding also contributes to adhesion between PEI and bio-tissues [13].

Hydrogel bioadhesives show reduced adhesion or even become ineffective in a wet environment because the existence of a thin hydration layer prevents interaction at the interface. This limitation spurred the exploration of other wet adhesion mechanisms, especially from a chemical viewpoint. Marine mussels have been extensively studied due to their impressive underwater adhesion to diverse surfaces, contributing to new generations of bioinspired materials [49,50]. It is found that six types of proteins (i.e., Mussel foot proteins (Mfps)) secreted by mussels polymerize to form the byssal thread and adhesive plaque to anchor to foreign wet surfaces [50]. These proteins have different molecular weights, but all contain the amino acid 3,4-dihydroxyphenyl-l-alanine (DOPA). Their independent but cooperative roles facilitate adaptive strong wet adhesion in even harsh environments [[50], [51], [52]]. The catechol side chain of DOPA also enables covalent crosslinking through its oxide (quinone) form or noncovalent crosslinking through chelation of metal ions, the latter endowing the mussel byssus with self-healing properties. Further, DOPA constitutes versatile noncovalent adhesive moieties interacting with diverse substrates, including bidentate hydrogen bonding, metal–catechol coordination bonding, π–π/π–cation interactions, and electrostatic interactions [51]. Distinct from their role in adhesion, Mfps are also important in controlling the stiffness and chemical stability (e.g., the protease resistance) of threads. For example, the high level of disulfide bonds in Mfp-6 helps to maintain adhesion by controlling the redox chemistry of DOPA and counteracting the vulnerability of DOPA to oxidation (i.e., DOPA-quinone) [51,53].

In light of its critical roles in biological organisms, DOPA and its derivatives were among the first adhesive moieties incorporated into synthetic hydrogel adhesives (i.e., mussel-inspired bioadhesives), including hyaluronic acid [54,55], PEG [56] and PAM [57,58], for enhanced adhesion. However, conjugated DOPA along the backbone of polymers tend to crosslink to increase the mechanical strength (cohesion). Since catechol groups in these bioadhesives are severely consumed during such crosslinking, mussel-inspired bioadhesives show low adhesion strength due to reduced catechol groups available for adhesion. Click chemistry has been introduced to citrate-based mussel-inspired bioadhesives as a secondary crosslink network to maintain the mechanical robustness of mussel-inspired bioadhesives [59] (Fig. 2b) through the reaction of alkyne and azide moieties, forming rigid triazole rings while enhancing adhesion strength. As a result, these mussel-inspired bioadhesives show high adhesion strengths of up to 223.11 ± 15.94 kPa, which is 13 times higher than that of commercially available fibrin glue. Another strategy to achieve higher adhesion without compromising cohesion in DOPA-conjugated PAM hydrogels has been demonstrated in Ref. [58], where mesoporous silica nanoparticles were incorporated as a bridge between DOPA and PAM through hydrogen bonding.

The introduction of DNs has proven to be an effective method to achieve various drug delivery and curative effects curative of hydrogels to allow the transition from predominantly passive wound dressings to actively regenerative biomaterials [60,61]. External hydrophilic antibiotics or drugs can be easily loaded into hydrogel networks due to their intrinsic hydrophilicity. By the incorporation of hydrophobic moieties or nanoparticles in hydrogel networks, hydrophobic drug loading and delivery can also be achieved [62,63]. By introducing dendritic nanogels (DNGs) with hydrophobic cores into a polyethylene glycol (PEG) network through ultraviolet (UV) curing, both hydrophilic (novobiocin sodium salt, NB) and hydrophobic drug (ciprofloxacin, CIP) loading and controllable delivery were achieved in a single hydrogel bioadhesive [63] (Fig. 2c-ⅰ). The accumulative release of hydrophilic NB was nearly 100% over 4 h due to the high swelling ratio of hydrogels (Fig. 2c-ⅱ), while the CIP release was slowed down by the hydrophilic PEG network due to the barrier effect (Fig. 2c-ⅲ). As a result, increasing the weight ratio of PEG leads to a slow release of CIP. The synergistic effect from CIP and NB leads to an enhanced bacterial killing effect compared with a single-component release (CIP or NB). Further, controllable drug delivery of hydrogel bioadhesives with specific release profiles can be achieved by changing the polymer-polymer or polymer-water interaction. In addition to the diffusion process, the swelling or de-swelling state of hydrogels in response to multiple stimuli (e.g., temperature, pH value, and light) makes it suitable for controllable drug delivery [[64], [65], [66]].

### 3.2 Immunomodulatory hydrogels

**Fig. 3:**
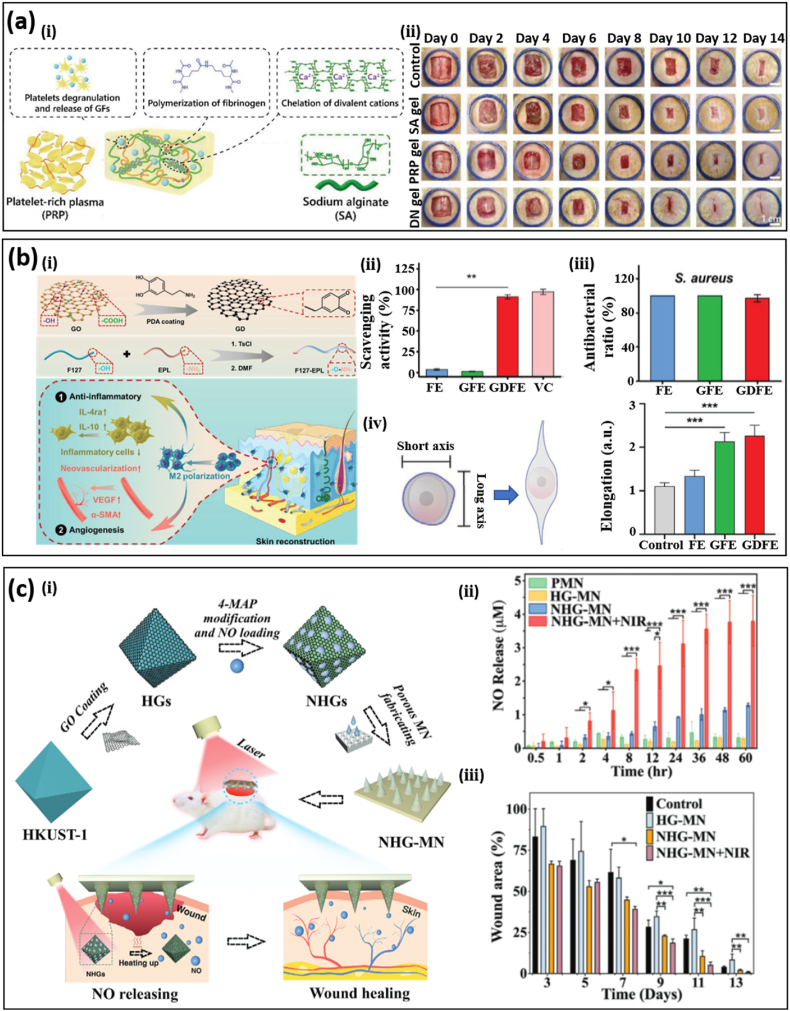
Immunomodulatory hydrogel bioadhesives. (a) (i) An immunomodulatory hydrogel bioadhesive based on a dual network of platelet-rich plasma and sodium alginate. **(ii)** Comparison of re-epithelialization performance in a rat model with treatments by blank, single-network (SA or PRP gel), or dual-network hydrogel bioadhesives. The improved mechanical property and release of growth factors in the dual-network hydrogel bioadhesive compared to the individual components (i.e., sodium alginate and plasma) contribute to enhanced wound healing. Reprinted with permission from Ref. [35]. **(b) (ⅰ)** An immunomodulatory F127 hydrogel bioadhesive with DOPA-modified graphene oxide (GO) and ε-polylysine (EPL) for simultaneous antibacterial and immunoregulation. Scavenging activity **(ⅱ)**, antibacterial **(ⅲ)**, and M2 phenotype polarization **(ⅳ)** of the GDFE hydrogel bioadhesive, compared to that with a single component (i.e., FE and GFE) and the control group. VC denotes the ascorbic acid group. Reprinted with permission from Ref. [12]. **(c) (ⅰ)** Hydrogel bioadhesives with poly (ethylene glycol) diacrylate (PEGDA)-based porous microneedle (MN) structures loaded with nitric oxide delivery. Nitric oxide was loaded in a copper-benzene-1,3,5-tricarboxylate (HKUST-1) metal-organic framework (MOF). Photothermal responsive graphene oxide was used to encapsulate HKUST-1 to obtain controllable nitric oxide release. Nitric oxide release profile **(ⅱ)** and wound closure in a diabetic rat model **(ⅲ)** among different groups, where PMN, HG-MN, NHG-MN, and NHG-MN + NIR stands for pure PEGDA-MN, PEGDA-MN with HKUST-1@GO loadings, PEGDA-MN with NO@HKUST-1@GO loadings, PEGDA-MN with NO@HKUST-1@GO loadings upon near-infrared light irradiation. Reprinted with permission from Ref. [73].

As discussed in the previous section, wound healing is a highly orchestrated process with an innate and specific adaptive immune system. To be specific, the innate immune system detects foreign signals at the inflammatory step and the specific adaptive immune system then takes action to clear pathogens [4]. The signaling network composed of various growth factors, cytokines, and chemokines plays an important role in coordinating the immune response [67]. Interruption of the immune response will delay or prevent wound healing, possibly leading to amputation or death. For example, chronic wounds display a delayed transition from the pro-inflammatory to the pro-regenerative stage, manifested by abnormal expression of cytokines and excessive ROS. Therefore, triggering an inherent immune response through external stimuli in patients with severe infection or immune dysfunction can be an effective way to combat chronic wounds. Instead of loading drugs to battle against infection or inflammation, immunomodulatory hydrogels trigger the immune response from human bodies by incorporation of hydrogel chemistry, surface treatment, biological molecules, and stem cells [4,11,12,68,69]. Intuitively, plasma is an effective choice for immunomodulation, considering its constituent bioactive molecules, such as growth factors. However, the low mechanical stiffness and burst release of growth factors from plasma have severely restricted its applications in bioadhesives and chronic wound healing. A DN hydrogel bioadhesive based on fibrinogen from plasma and sodium alginate polymerized with thrombin and CaCl_2_ has been prepared through a simple polymerization process [35] (Fig. 3a-ⅰ). Due to the penetration of each network, the DN hydrogel bioadhesive shows a higher mechanical stiffness (∼300 Pa, 5 times that of the platelet-rich plasma gel) and a more sustainable release of growth factors. In an in vivo excision wound model, the release of platelets and exogenous proteins from the DN hydrogel bioadhesive more than doubled the production of transforming growth factor-β1 after 14 days, compared with the control group. A full-thickness wound in rats treated with the DN hydrogel bioadhesive corresponds to closure of ∼95% after 14 days, which is much higher than those treated solely with PRP (∼82%) or SA (∼84%) (Fig. 3a-ⅱ). Hydrogel chemistry, crosslinking density, and mechanical stiffness play an important role in triggering and modulating the human immune response. For example, dendritic cells treated with chitosan hydrogels can lead to higher expression of pro-inflammatory markers and cytokines compared with cells treated with alginate and agarose, possibly due to differences in cell adhesion [70]. Surface chemistry and topography of hydrogel bioadhesives can also control the immune response effectively [71]. Mesenchymal and adipose stem cells coated on hydrogel bioadhesives help to accelerate wound healing and mitigate scar formation as a result of the secretion of bioactive molecules [72]. Immunomodulatory hydrogels with various cellular and molecular signals have demonstrated polarization of macrophages from the inflammation to proliferation (M2) phenotype, which is considered to be key in chronic wound healing. An immunomodulatory F127 hydrogel with DOPA-modified graphene oxide (GO) and ε-polylysine (EPL) has been proposed for simultaneous immunoregulation and antibacterial effects [12] (Fig. 3b-ⅰ), remaining an injectable liquid at 4 °C and transitioning to gel state at 37 °C. The immunomodulatory hydrogel can effectively remove free radicals (Fig. 3b-ⅱ) and increase the antioxidant rate (Fig. 3b-ⅲ) due to the oxidation of DOPA to DOPA quinone. The effectiveness of the immunomodulatory hydrogel in promoting macrophage polarization to M2 phenotype was clearly shown in an in vitro experiment, manifested by a larger degree of cell elongation (∼2), compared to the control group (∼1) (Fig. 3b-ⅳ). The effectiveness of immunomodulatory hydrogels in diabatic wounds was manifested by a shorter wound length (30%) and thicker granulation tissue (150%) after 21 days.

Nitric oxide (NO) generated at the inflammation and proliferation stage plays an important role in the physiological and pathological process, including collagen formation, angiogenesis, and signal transmission between immune cells, rendering maintenance of a suitable nitric oxide concentration critical. Compared to other bioactive molecules, sustainable gas release is more challenging, especially for deep tissues. A porous metal-organic framework (MOF) microneedle array has been demonstrated for NO delivery upon photothermal actuation (Fig. 3c) [73]. NO was loaded in copper-benzene-1,3,5-tricarboxylate (HKUST-1) microparticles and then encapsulated by a photothermal responsive layer of GO (Fig. 3c-ⅰ). These microparticles were loaded in a poly(ethylene glycol) diacrylate (PEGDA)-based porous microneedle (MN) patch to realize on-demand release of NO through NIR irradiation. It was found that the MN patch loaded with NO@HKUST-1@GO (NHG) microparticle upon NIR irradiation (i.e., NHG-MN + NIR) corresponds to the highest cumulative release of NO (Fig. 3c-ⅱ) and wound closure in a diabetic rat model (Fig. 3c-ⅲ), compared with the pure PEGDA-MN (PMN), PEGDA-MN with HKUST-1@GO loadings (HG-MN), PEGDA-MN with NO@HKUST-1@GO loadings (NHG-MN).

The balance between the degradation rate of immunomodulatory hydrogels and tissue ingrowth rate is another important issue in wound healing, considering the critical role of scaffolding (i.e., physical support) in re-epithelialization, neovascularization, and formation of granulation tissue [68,74,75]. Previous research has demonstrated the efficacy of a peptide-based microporous annealed particle (MAP) scaffold in accelerating wound healing due to the fast formation of a 3D cell network in the scaffold [74]. A recent study aimed to decrease the scaffold degradation rate and increase tissue ingrowth by switching the chirality of the crosslinking peptides from L-to d-amino acids [68]; however, D-MAP degradation in vivo in a murine splinted excisional wound model was not decreased, possibly due to enhanced myeloid cell recruitment from the immune response. Even though both L- and D-peptides can trigger the innate immune response, including the myeloid cell and macrophage recruitment, the additional adaptive immune response to D-peptides leads to fast degradation and enhanced skin regeneration, air neogenesis, and improved tensile strength of neogenic tissues.

### 3.3 Injectable hydrogels

**Fig. 4:**
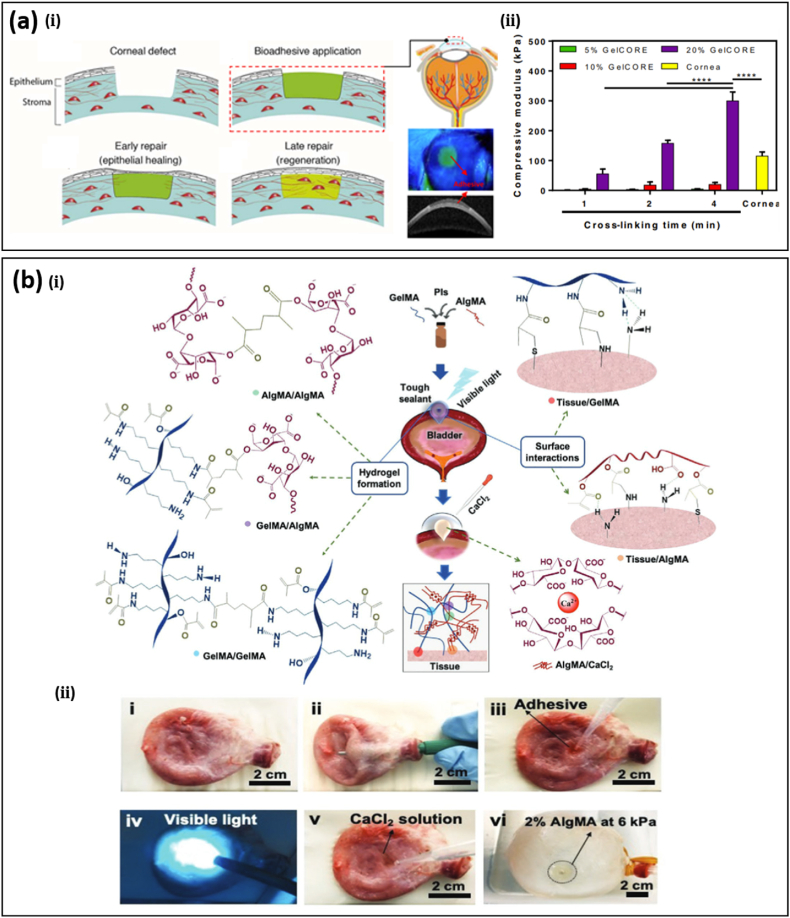
Injectable hydrogel bioadhesives. (a) (i) Schematic illustration of the treatment of corneal defects by gelatin-based and visible light-curable hydrogel bioadhesive. **(ii)** Tunable mechanical stiffness of the gelatin-based bioadhesive by changing the crosslinking time and prepolymer concentration to match that of the cornea for improved sealing ability, stromal regeneration, and re-epithelialization. Reprinted with permission from Ref. [15]. **(b) (i)** Schematic illustration of an injectable hydrogel bioadhesive with ultrastretchability due to the effective energy dissipation by Ca^2+^-induced reversible crosslinking. **(ii)** Sealing a bladder defect with the injectable hydrogel bioadhesives followed by photocrosslinking and CaCl_2_ treatments leads to a burst pressure of 6 kPa. Reprinted with permission from Ref. [14].

Injectable hydrogel bioadhesives with good flowability and sealing ability can post-polymerize with additives [13,76] or under external stimuli (e.g., optical [14,15,77,78], pH [79], and thermal [80]) after being applied to wounds, thus offering tremendous advantages in internal or irregular wound healing with limited external accessibility. A suitable viscosity of precursors is beneficial for such wounds, including gastric, corneal, and bladder. A DN hydrogel with polydextran aldehyde (PDA) as the adhesive component and PEI as the crosslinker can be applied in an injectable form with post-polymerization at room temperature [13]. The resulting DN hydrogel bioadhesive shows a maximal adhesive strength of ∼2.8 kPa while the antimicrobial effectiveness was supported by more than 90% reduction of *E. coli* and *S. aureus*. In contrast to the control group, wounds in a murine model treated with the PDA/PEI bioadhesive displayed neither abscesses nor inflammation. Injectable hydrogel bioadhesives have been demonstrated in the treatment of corneal injuries where traditional suture application is difficult due to limited working space and tissue sensitivity [15] (Fig. 4a-ⅰ). Based on methacrylic anhydride modified gelatin and visible-light photoinitiator, a transparent hydrogel bioadhesive with tunable mechanical properties, swelling ratio, and biodegradability has been obtained by changing the polymer concentration and photocrosslinking time. For example, increasing the photo-crosslinking time from 1 to 4 min leads to the increase of its compressive modulus from 1.2 to 4.5 kPa (Fig. 4a-ⅱ). With 20% gelatin and a crosslinking time of 2 min, the obtained hydrogel bioadhesive has a similar stiffness (∼200 kPa) to the native cornea (∼130 kPa). The water content of the hydrogel bioadhesive remains in the range from 85.8 to 89.5% with different photo-crosslinking times, which also matches that of human corneas. In vivo assessment of the hydrogel bioadhesive in a rabbit stromal defect model shows that it can form a firm transparent layer on the corneal defect immediately after crosslinking (within 4 min). Compared to the untreated group, faster migration of the epithelium over the bioadhesive hydrogel was observed, leading to the recovery of the corneal defect after 14 days of application, while the untreated control maintains an uncovered stromal layer. In some cases, injectable bioadhesives with ultra-stretchability are needed for wounds in organs under large and cyclic deformations, such as the lung, heart, and bladder. A photocrosslinkable hydrogel with ∼600% stretchability based on gelatin and alginate functionalized with vinyl groups (i.e., gelatin methacryloyl and methacrylate-modified alginate, GelMA and AlgMA) to seal bladder wounds has been proposed (Fig. 4b) [14]. The conjugated methacryloyl (MA) group can be activated upon the use of photoinitiators. Ultra-stretchability originates from efficient energy dissipation in the hydrogel bioadhesive through reversible electrostatic interactions between calcium ions and the α-L-(1–4)-guluronate residue (G blocks) in AlgMA. Covalent bonding between MA groups of GelMA and AlgMA contributes to more than 600% improved toughness compared to GelMA (Fig. 4b-ⅰ). The burst pressure of a porcine bladder with annular perforation increases from 2 to 5 kPa by applying the photocrosslinkable hydrogel with 2% AlgMA (Fig. 4b-ⅱ).

### 3.4 Hydrogels with nanomaterial loadings

Recently, the introduction of novel nanomaterials has endowed hydrogel bioadhesives with unique electrical [81,82] and optical [83] properties, enabling direct interfacing with biosensing or physical therapies for wound healing. Conductive bioadhesives have been proposed to enable the direct implementation of bioelectronic devices on wounds. In one representative study, the addition of graphene into a poly(vinyl alcohol) hydrogel with an interpenetrating crosslink network of poly(acrylic acid) grafted with *N*-hydroxysuccinimide ester (PAA-NHS ester), resulted in a conductive bioadhesive maintaining a high conductivity (>2.6 Sm^−1^) during 14-day incubation in phosphate-buffered saline solution [81]. Concomitantly, the formation of covalent crosslinks with primary amine groups led to strong tissue adhesion, allowing intimate interfacing between tissues and bioelectrodes for biopotential collection and electrical stimulation. By attaching gold electrodes on a rat heart with conductive bioadhesive as a middle layer, the epicardial electrocardiogram (ECG) signal was collected with comparable signal-to-noise ratios to surface ECG over14 days of continuous measurement. Periodic and stable ankle joint movements were observed under electrical stimulation of rat sciatic nerves. The efficacy of conductive bioadhesives in physiological potential measurement and electrical stimulation implies its potential in electrical stimulus for wound healing [84]. In another work, the optical absorption of bioadhesives can be tuned by introducing nanomaterials for the photothermal treatment of chronic wounds [83]. Based on the photothermal effect of biodegradable black phosphorus (BP) nanosheets, controllable anesthetic (lidocaine hydrochloride) delivery by hydrogel bioadhesives has been achieved upon near-infrared light (NIR) irradiation. Upon NIR laser irradiation with a wavelength of 808 nm and a power of 1 W cm^−2^ for 5 min, wound temperature increased by 26.5 °C, much higher than when treated by NIR irradiation alone or the BP-based hydrogel in the absence of irradiation (∼5 °C). Rapid heating enables on-demand antibacterial effects and pain relief, improving antibacterial activity, cell proliferation, vascularization, and angiogenesis. In vivo experiments with both BP hydrogel and NIR treatments demonstrate the smallest wound area in a diabetic murine model after 10 days (5.44, 2.25, and 2.23 times smaller than that in the control group, treated by the BP-free bioadhesive or the BP hydrogel without NIR irradiation).

## 4 Bioadhesives with mechanical and/or electrical stimulation

**Fig. 5:**
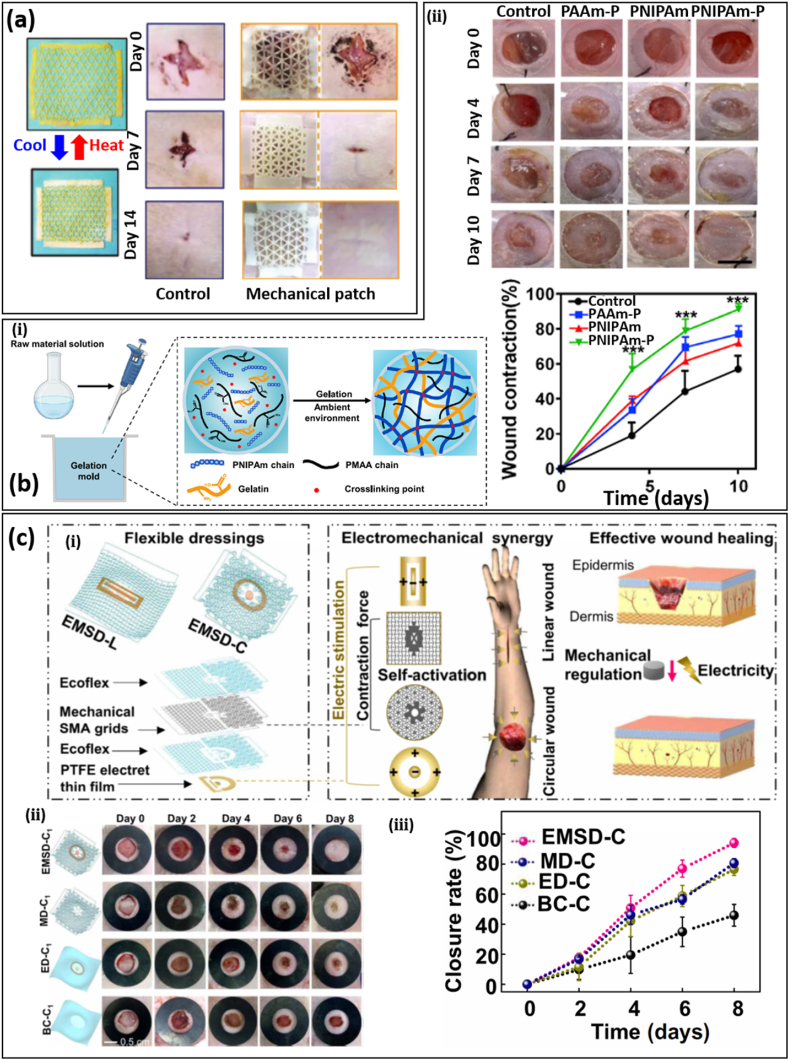
Bioadhesives with mechanical and/or electrical stimulation. (a) (ⅰ) A mechanically active bioadhesive based on liquid crystal elastomers with significant biaxial thermal shrinkage and low actuation temperature (46 °C) for wound healing. **(ⅱ)** Optical images of the skin wound healing without treatments or treated by the mechanical active bioadhesive. Reprinted with permission from Ref. [86]. **(b) (i)** A mechanically active and immunomodulatory bioadhesive based on temperature-sensitive poly(*N*-isopropylacrylamide) (PNIPAm) and poly(methacrylic acid) PMAA. optical images of wounds **(ⅱ)** and measured wound closure **(ⅲ)** in diabetic mice treated with non-temperature-sensitive hydrogel (PAAm), immunomodulatory hydrogel (PNIPAm), and mechanically active and immunomodulatory hydrogel (PNIPAm-P). Reprinted with permission from Ref. [69]. **(c) (ⅰ)** Schematic illustration of an electromechanical synergistic dressing (EMSD) composed of a shape memory alloy (SMA)–based mechanical metamaterial grid and a polytetrafluoroethylene (PTFE) electret electrostatic film (EEF) for linear (L) or circular (C) wounds. Optical images **(ii)** and wound closure rate **(ⅲ)** of a circular wound in different groups over 8 days' observation. EMSD-C, MD-C, ED-C, and BC-C correspond to the simultaneous mechanical and electrical stimulation, solely mechanical or electrical stimulation, and blank control. Reprinted with permission from Ref. [87].

The effect of stretching on tissue regeneration has been studied at single-cell resolution, where it was found that basal cells respond to stretching through transcriptional regulators (such as formin-like proteins and non-muscle myosin) [85]. Mechanical loading has also been demonstrated as an effective way to accelerate wound healing, especially for large and open wounds [69,86]. Shrinkage of hemostatic patches loaded with shape memory polymers/alloys or liquid crystal elastomers can be triggered by temperature, light, or pH due to the existence of two or more phases of these materials. In a representative study, a metamaterial consisting of horseshoe microstructures based on a liquid crystal elastomer offers a biaxial strain of ∼53% at a low actuation temperature (46 °C) (Fig. 5a) [86]. In vivo animal experiments show that the shrinkable hemostatic patch improves wound recovery by more than 30%, compared with the control group. This technique can be easily integrated with multiple sensing systems, such as temperature and pH, for real-time modulation of wound healing. In another representative study, the synergistic effect of mechanical loading and immunomodulation has been demonstrated in a temperature-sensitive hydrogel bioadhesive (Fig. 5b-ⅰ) [69]. Gelatin as the adhesive functional component and poly(methacrylic acid) (PMAA) as the immunomodulatory component were added to a poly(*N*-isopropylacrylamide) (PNIPAm) network through free radical polymerization. PMAA has been used in modulating the polarization of macrophages, controlling cytokine secretion, and promoting tissue ingrowth. Due to the phase transformation, the pore size of PNIPAm hydrogels decreases by more than 50% when the temperature increases from 25 to 37 °C. In an in vivo mouse model, greater wound closure (∼90%) after 10 days was observed for PNIPAm hydrogels versus the control group (Fig. 5b-ⅱ and **ⅲ**). A synergistic effect from mechanical and electrical stimulation in wound healing has been demonstrated in an electromechanical synergistic dressing (EMSD) composed of a shape memory alloy (SMA)–based mechanical metamaterial grid and a polytetrafluoroethylene (PTFE) electret electrostatic film (EEF) (Fig. 5c-ⅰ) [87]. The mechanical metamaterial grid can be fabricated to obtain proper contraction forces for wounds with different shapes, such as circular or linear. The EMSD was stretched by 10% before applying on wounds and underwent contraction due to the phase change triggered by skin temperature (30–35 °C). Patterned PTFE EFTs with the inner (outer) part being positively (negatively) charged were used to obtain electrical stimulation. Full-thickness circular skin wound treated by EMSD (EMSD-C) shows the highest closure rate over continuous 8 days’ observation (Fig. 5c-ⅱ). The EMSD-C group corresponds to a closure rate of 96.8% on day 8, which is significantly higher than the control group (BC–C) and that treated by solely mechanical (MD-C) or electric (ED-C) stimulation (Fig. 5b- ⅲ). It was further confirmed that the accelerated wound healing is a result of the enhanced secretion of VEGF and transforming growth factor–β (TGF-β) from mechanical contraction and electric stimulation.

## 5 Smart wound healing

### 5.1 Biosensors for wound monitoring and evaluation

Wound healing is a dynamic process, thus real-time monitoring of the wound status for feedback is essential for recovery [[88], [89], [90]]. This is especially true for chronic wounds (e.g., venous, diabetic, and pressure ulcers) which usually cannot regenerate within 3 months. Due to potential infection and prolonged inflammation, patients with chronic wounds need to travel to the hospital frequently for diagnoses and treatments, such as debridement or dressing changes. Currently, wound evaluation is mainly based on clinicians’ visual inspections and experience, which is highly subjective and may lead to incorrect conclusions. With the development of technology, flexible biosensors play an important role in the management of chronic wounds by continuously monitoring and assessing wound status. Sensing data can provide useful information to help doctors diagnose chronic wounds without removing the bandage (reducing the burden on patients), and further develop optimal treatment strategies throughout the healing process. According to related research, the pH of wound fluid, wound temperature, uric acid (UA), glucose, oxygen, and moisture levels, have been recognized as important diagnostic parameters for assessing the condition of chronic wounds.

The pH value is an essential parameter that can notably influence many physiological processes such as collagen formation, inflammatory response, and angiogenesis. For acute wounds, the pH value is in the range of 4–6 with the slight acidity preventing bacterial colonization; however, chronic wounds are more alkaline with pH values oscillating between 7 and 9 due to the improper immune response, leading to their susceptibility to infection following bacterial incursion and colonization within wound sites [91,92]. Therefore, pH monitoring of chronic wounds is an effective method to predict the healing phase and provide a forewarning of infection risks.

Uric acid is mainly present in wound exudate in the form of urate, with a concentration ranging from 220 to 750 × 10^−6^ _M_ [93]. Because the human body lacks a specific enzyme, UA cannot be catabolized inside the body and is physiologically excreted, mainly through urine. Increased UA levels in body fluids caused by diseases including diabetes result in gout and urate crystal precipitation in joints, kidneys, and other tissues. In contrast, certain bacteria, such as *Pseudomonas aeruginosa*, can specifically metabolize UA, decreasing UA concentration in the wound exudate to below 200 × 10^−6^ M [93]. Therefore, UA levels in skin wounds can serve as an effective indicator of bacterial infection.

Glucose level is also a crucial indicator of chronic wound (especially for diabetic wound) status and the physical condition of patients. Cytokines are mainly regulated by hypoxia through the transcription factor hypoxia-inducible factor-1 (HIF-1), which consists of α and β-subunits (HIF-1α and HIF-1β). It plays an important role in the regulation of cell oxygen homeostasis [94,95]. HIF-1a is highly expressed in wound beds during the early stage of normal wound healing; however, it is impaired in diabetes due to high glucose concentration, which results in the insufficient formation of capillary blood vessel networks, and further leads to tissue necrosis [96]. Therefore, variation in wound glucose level can reflect wound condition and its monitoring is therapeutically significant to guide the clinical treatment of chronic wounds [97,98].

Temperature is regarded as the most significant factor for the assessment of chronic wounds as: (1) the healing process involves a series of chemical and enzymatic actions and normal temperature is necessary for all enzyme and cell functions within these actions, (2) body temperature could affect local blood flow and lymphocyte extravasation [90], and (3) temperature may influence other wound characteristics (e.g. inflammation) [99]. Several studies show that the activities of fibroblasts, neutrophils, and epithelial cells will decrease and further impair wound healing when the temperature is below 33 °C, whereas reduction of ulcer area is promoted while the temperature is in the range of 36–38 °C [100,101]. Therefore, monitoring of temperature can prevent ulcers and predict the occurrence of infection, and abnormal temperature changes can be used as a predictor in the early stages of infection before other obvious symptoms [17,102].

Oxygen is also considered a key factor in promoting wound healing. The human body needs oxygen to enable bacterial defense, cell proliferation, collagen synthesis, and angiogenesis in the process of wound healing [90]. For example, blood vessels around the wound sites help to deliver fresh nutrients and oxygen to the wound to promote healing. The higher oxygen content can trigger white blood cell macrophages (the transparent liquid surrounding the wound) to enter the wound, fight infection, and send growth factors to heal the wound [90], while the main function of oxygen is its ability to generate chemical energy by promoting oxidative metabolism, which enables the reproduction of injured cells. In contrast, hypoxia can slow down or even stop the process of wound healing. Therefore, it is valuable to monitor oxygen content during the wound healing process.

Wound infections promote the generation of secretions, which in turn increase the moisture level of chronic wounds. The increased moisture will cause bacteria to proliferate and increase the risk of infection [103]. Furthermore, moisture-induced wound infections can increase tissue fluid and cause the wound to become excessively wet, thereby increasing the risk of secondary infection [104] in a cyclical process. Moisture balance is important in achieving optimum wound healing conditions because a wet wound can lead to maceration whereas too little moisture will lead to desiccation. Moisture imbalances, therefore, necessitate dressing changes; however, it is difficult for clinicians to observe the moisture status of the wound without disturbing traditional dressings, which is very likely to cause unnecessary replacement (every 1–3 days), wasting manpower and resources or insufficient replacement, resulting in wound infections [105]. For example, a study conducted by Milne et al. found that about 44.9% of 588 patients had wound moisture in the optimal range (two to four drops measured by moisture meter) during dressing change [105], which indicates a large number of unnecessary dressing changes. Therefore, in order to prevent further deterioration of wound infections and tissue necrosis, it is crucial to monitor moisture during wound healing.

The above sections amply demonstrate that monitoring wound conditions (e.g., UA, pH, temperature, moisture, glucose, and oxygen) is critical to early identification and management of chronic wounds. It has additionally been noted that monitoring of a single physiological condition is not enough to lead to an insightful and correct evaluation of wound conditions. In order to fully evaluate wound conditions, it is highly desirable to integrate multiple biosensors into bioadhesives for practical applications [90,93,103,[106], [107], [108], [109], [110], [111]]. In the following, bioadhesives with multifunctional sensing modules for chronic wound monitoring will be discussed.

**Fig. 6:**
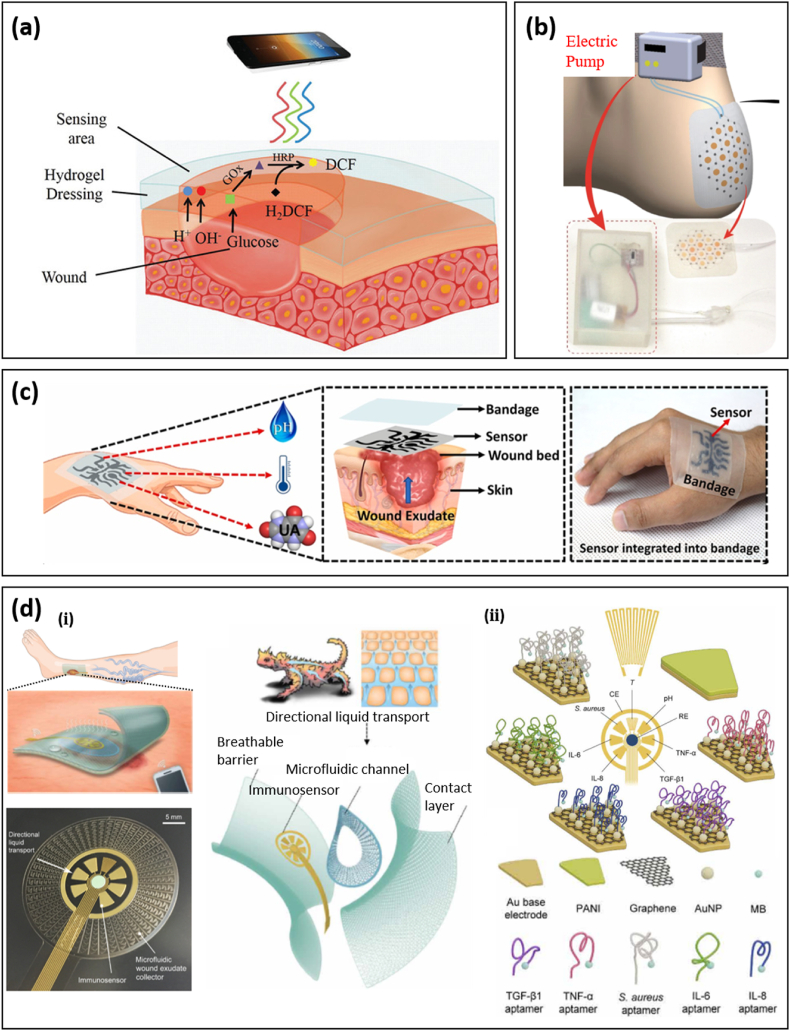
Integrated biosensors for the real-time evaluation of wounds. (a) Scheme of zwitterionic PCB hydrogel dressing for the detection of pH value and glucose concentration in wound exudate. Reprinted with permission from Ref. [111]; Copyright 2020, John Wiley & Sons, Inc. **(b)** Overview illustration of smart dressing integrated with oxygen sensing and delivery patch. Reprinted with permission from Ref. [112]. **(c)** Schematic illustration of a stretchable and flexible smart bandage integrated with UA, pH, and temperature sensors, and a photograph of this device adhered on the hand for monitoring the condition and status of the wound. Reprinted with permission from Ref. [113]; Copyright 2020, Elsevier. **(d) (ⅰ)** A flexible multiplexed immunosensor integrated with a bio-inspired fluidic sampling system based on inter-connected half-open, sawtooth-shaped capillary channels with decreasing width, and a flexible wireless module for real-time monitoring of venous ulcers. **(ii)** Electromechanical sensing of cytokine and bacteria was based on aptamer modified graphene gold electrodes. Reprinted with permission from Ref. [18].

For diabetic ulcers, a multifunctional zwitterionic poly-carboxybetaine (PCB) hydrogel wound dressing that includes a pH indicator dye and two glucose enzymes was proposed to simultaneously monitor the pH and glucose level as shown in Fig. 6a [111]. This device has good stability and sensitivity within the pH range of 4–8, realized by incorporating phenol red as a pH-sensitive, non-toxic dye, and within glucose levels of 0.1–10 × 10^−3^ M, achieved by encapsulating glucose oxidase and horseradish peroxidase. Hydrogel color change from yellow to red with the increase of pH values can be split into red, green, and blue (RGB) and imaged via smartphone and handled with analytic software (MATLAB) to read the RGB data. For glucose measurements, PCB hydrogel discs dipped with different concentrations of glucose were observed under 365 nm UV light, demonstrating enhanced fluorescence intensity with the increase of glucose concentration from 0–10 × 10^−3^_M_. A microplate reader was utilized to establish a quantitative curve between glucose level and fluorescence intensity. As a result, the pH value and glucose level can be monitored precisely to provide useful information for clinical intervention and wound management. In addition to dual monitoring capabilities, the dressing achieved enhanced healing compared to commercial DuoDerm dressings, with approximately 88% and 70% wound area reduction after 8 days, respectively.

Chronic wounds lack a functional vascular network and are therefore unable to receive enough oxygen to promote healing. In order to solve this problem, a paper-based wearable, flexible, and biocompatible smart wound dressing was proposed [112]. This smart platform comprises an oxygenation patch and oxygen-sensitive ink, which can provide continuous oxygen delivery and optical sensing in wound regions (Fig. 6b). This patch consists of a flexible microfluidic network bonded to a substrate made of active parchment paper. The parchment paper is regarded as a hydrophobic structural and functional material; however, its surface energy is tunable by plasma for increased hydrophilicity and ink adhesion. The natural mesh structure of paper allows embedding with chemicals suspended in an aqueous solution. In this smart wound dressing, oxygen generation and delivery are achieved by flowing H_2_O_2_ with an electric pump into the microchannel network, where the H_2_O_2_ is decomposed by a catalyst to generate oxygen when reaching catalyst-containing regions, followed by oxygen diffusion into the wound bed. Oxygen sensing is realized by oxygen-sensitive ink based on a ruthenium compound, which exhibits a sufficiently strong phosphorescent response to be detected using an external optical probe. As a result, the designed smart dressing can increase oxygen concentration 25–50% above ambient conditions in 1 h and is able to sense oxygen in a range of 5–26 ppm, achieving simultaneous delivery and sensing.

A wearable smart wound bandage integrated with pH, UA, and temperature sensors was designed to monitor these three parameters simultaneously as shown in Fig. 6c [113]. For this smart bandage, researchers applied 2D multilayered nanosheets (MXenes) to functionalize 3D laser-guided graphene (LGG) sheets via C–O–Ti covalent crosslinks to obtain an LGG-MXene hybrid scaffold. This strategy gives the hybrid scaffold high conductivity, overcoming the high sheet resistance and poor mechanical properties of LGG sheets. Furthermore, improved electrochemistry is achieved, with a fast heterogeneous electron transfer rate due to the synergistic effect between LGG and MXene. The final smart, flexible, and stretchable multifunctional sensors-integrated wound bandage is fabricated by transferring the hybrid scaffold onto a PDMS substrate. This integrated device exhibits excellent performance in three aspects: (1) the UA sensor shows rapid response toward UA in the extended range of 50–1200 μM with an excellent regression coefficient (R^2^) and sensitivity of 0.999 and 422.5 μA mM^−1^ cm^−2^; (2) the pH sensor exhibits a linear response with a high sensitivity of −57.03 mV pH^−1^ and regression coefficient (R^2^) of 0.998, respectively, in the wound relevant pH range of 4–9; and (3) the temperature sensor responds rapidly to physiological temperature variations in the range of 25–50 °C with an excellent sensitivity of 0.09% °C^−1^ and stable linear resistive response with a correlation coefficient of 0.999.

Since extreme/prolonged inflammation is the origin of chronic wounds and scar formation, further efforts have been made to measure inflammatory mediators to precisely evaluate the wound healing stage [18,114,115]. In a representative study, a microfluidic wound exudate collector inspired by the skin of the Texas horned lizard has been integrated into a multiplexed immunosensor patch to accurately monitor inflammatory mediators (including tumor necrosis factor–α (TNF-α), interleukin-6 (IL-6), IL-8, transforming growth factor–β1), bacterial load (*Staphylococcus aureus*), and physiological parameters (temperature and pH) (Fig. 6d-ⅰ) [18]. A flexible wireless module was integrated with the sensing patch to allow for real-time monitoring of venous ulcers. To counteract the small amount of wound exudate, an efficient fluid sampling system based on interconnected half-open, sawtooth-shaped capillary channels with decreasing width was introduced to the sensing patch. Aptamers corresponding to the desired inflammatory mediators were dropped onto graphene-gold nanoparticles to obtain modified working electrodes for specific sensing (Fig. 6d-ⅱ). The concentration of biomarkers was correlated to the peak height in square wave voltammetry measurements. Obtained biomarker profiles can provide insightful and comprehensive wound-specific parameters for clinical decisions.

### 5.2 Multifunctional or closed-loop bioadhesives

**Fig. 7:**
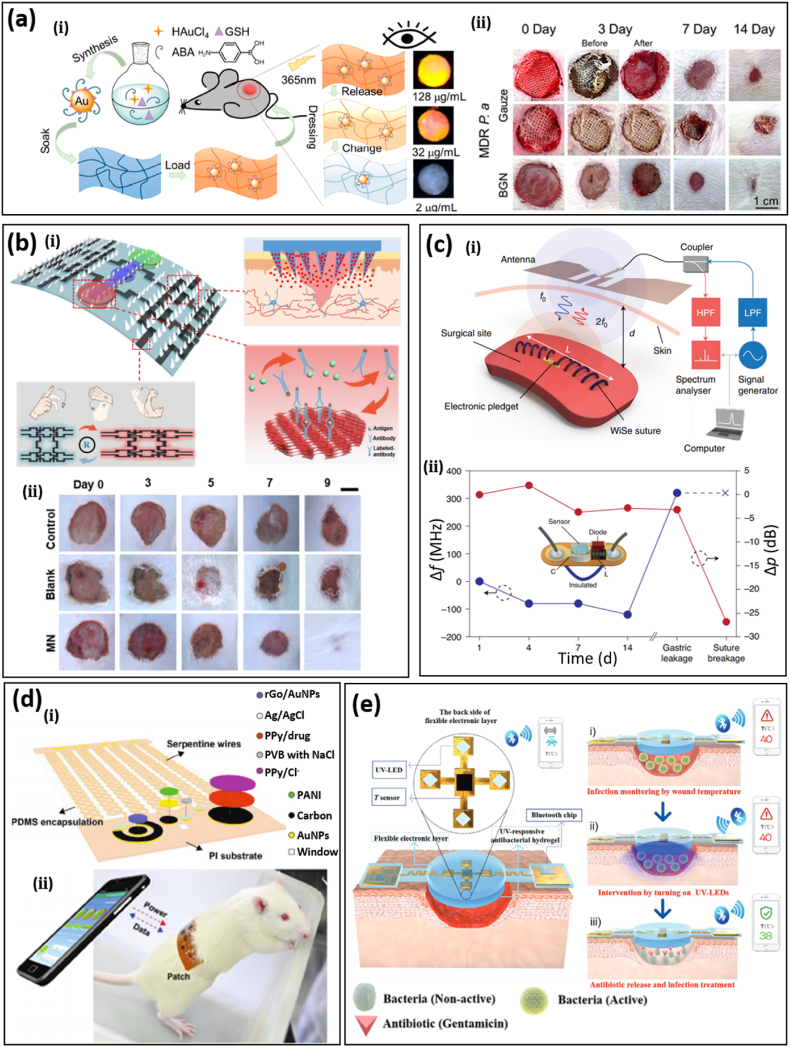
Smart bioadhesives with biosensing, treatments, and wireless modules. (a) (i) A smart bioadhesive with antibacterial properties based on Aminobenzeneboronic Acid (ABA) modified gold nanoparticles and self-monitoring of residual nanomedicine through fluorescence strength. **(ii)** Optical images of multi-drug-resistant (MDR) *Pseudomonas aeruginosa*-infected skin wound models on rats treated by gauze or the smart bioadhesive with/without debridement on day 3. Reprinted with permission from Ref. [117]. **(b) (ⅰ)** A bio-inspired multifunctional bioadhesive with drug delivery by microneedles, biosensing based on microfluidic channels for sampling and inverse opal photonic crystal for sampling and detection, and movement detection by MXene-based strain sensors. **(ⅱ)** Optical images of a foot wound in a diabetic mouse with different treatments. MN stands for the microneedle bioadhesive. Reprinted with permission from Ref. [119]. **(c) (ⅰ)** Wireless sensing based on a loop antenna composed of conductive sutures and an inductor-capacitor (LC) circuit on pledgets. The received RF signal by the loop antenna generates a second harmonic backscattered signal whose resonant frequency and magnitude indicate wound leakage and suture breakage, respectively. **(ⅱ)** The measured resonant frequency and magnitude when the wireless sensing system was applied on muscle over 14 days. Reprinted with permission from Ref. [26]. **(d) (i)** A closed-loop bioadhesive with the integration of biosensing (pH, uric, and temperature) and antibacterial drug release (i.e., cefazolin). **(ii)** Schematic diagram of the smart wound dressing working in the NFC wireless mode for data communication and powering. Reprinted with permission from Ref. [22]. **(e) (ⅰ)** Schematic of the structure of the integrated smart wound dressing consists of an electronic layer that consists of a temperature sensor and four UV-LEDs, a layer of UV-responsive antibacterial hydrogel, and a Bluetooth chip. The temperature sensor is used to monitor wound temperature, the UV-LEDs are used to emit UV light for the release of antibiotics from the UV-responsive antibacterial hydrogel, and the Bluetooth chip is used for wireless data transmission in real-time. **(ⅱ)** The local temperature rise at wounds was detected and transmitted to a cellphone through Bluetooth. The infection risk triggered the release of antibiotics from the UV-responsive hydrogel by turning on UV-LEDs. The local temperature recovers to normal after treatments. Reprinted with permission from Ref. [121].

Recent research has demonstrated the great advantage of integration of sensing and treatment through smart bioadhesives [116]. Physiological conditions at wounded sites measured by biosensors can be used to activate corresponding treatments to avoid infections and prolonged inflammation and reduce pain levels during dressing removal and subjective physical examination, mediated by the fast development and great success of the closed-loop system. Due to antibiotic abuse, multi-drug-resistant (MDR) bacterial infections have become a common issue in wound healing. Inorganic nanomaterials, such as gold/silver, zinc oxide, and titanium dioxide nanoparticles, have emerged as new antimicrobials to address this issue. Particularly, gold (Au) nanoparticles have been widely adopted in medical and clinic applications due to their biocompatibility and low toxicity. In a recent study, gold nanoparticles modified by aminobenzeneboronic acid (ABA) were coated on cellulose bioadhesives with self-monitoring capability for MDR bacteria-infected wounds (Fig. 7a-ⅰ) [117]. Surface modification of ABA on Au nanoparticles enables emission of bright orange fluorescence under UV light without compromising its antibacterial performance, with the intensity of ABA-modified gold nanoparticles under 365 nm light excitation increasing with concentration. This colorimetry-enabled direct monitoring of residual nanomedicine indicates the proper time for bioadhesive replacement. After 14 days, a *Pseudomonas aeruginosa*-infected wound treated by the nanomedicine attained 91% healing, compared with 75% for the control group (Fig. 7a-ⅱ). In another study, the color change based on microstructures in an adhesive patch has been utilized as an indicator of the mechanical deformation of tissues [118].

It has been found that most smart wound dressings with biosensing capabilities can't be applied directly due to inefficient wound exudate sampling, especially for trace molecules. Considering the effectiveness of microfluidic channels in sweat sampling, they have been integrated into a multiplexed immunosensor for wound monitoring. A tapered microchannel with sawtooth-shaped microstructures promotes forward flow while inhibiting reverse flow [18]. This directional liquid transport design leads to an additional 180% wound fluid collection within 130 s. Conventional drug delivery by hydrogel bioadhesives via passive diffusion is inefficient due to the tissue barrier. Microneedles can penetrate wounded tissues with minimal invasion, making them suitable for drug delivery [73,119,120]. A multifunctional and biomimetic bioadhesive integrating microneedle-enabled drug delivery, microfluidic channels, and MXene-based strain sensors has been designed for complete wound healing (Fig. 7b-ⅰ) [119]. Inspired by shark teeth, SF-based microneedles were found to greatly enhance adhesion. Further, a porous microneedle surface was obtained by etching SiO_2_-coated SF to increase drug loading for extended delivery. Wound exudate can flow through microfluidic channels due to capillary force and arrive at inverse opal photonic crystal detection areas. As a result, wound-related biomarkers, such as lactate and calprotectin, can be determined by fluorescence intensity. In a diabetic mouse model, the microneedle bioadhesive was loaded with epidermal growth factor for the treatment of foot ulcers (Fig. 7b-ⅱ), enabling a much higher recovery rate (the average recovery area for the microneedle group is ∼75%, while that for the control group is ∼30%). It was also found that the extended and controllable drug delivery via porous microneedles aids the regulation of inflammatory factors, such as IL-6 and TNF-α.

Wireless technology enables wireless sensing, data communication, and remote manipulation; thus, it is anticipated to play an important role in future smart wound healing. Conventionally, sensing data measured by biosensors attached to wounds is sent to external devices by wired connections, which severely restricts movement. Conversely, wireless sensing based on the change of resonant frequency and magnitude of antennas can be achieved using a remote interrogator, leading to a reduced form factor. In addition, passive sensing eliminates the need for batteries, which are unsuitable for deep internal wounds. In a representative study, a remote wireless sensing system based on a loop antenna composed of conductive sutures and an inductor-capacitor (LC) circuit on pledgets has been demonstrated for deep surgical wounds (Fig. 7c-ⅰ) [26]. The conductive suture was prepared by doping a biocompatible polymer (PEDOT: PSS) on silk filaments. The resulting suture shows a small variation of conductivity upon mechanical bending or being immersed in phosphate-buffered saline solutions. The received RF signal generates a second harmonic backscattered signal which can be detected by the external wireless system. When being applied to an incision, gastric leakage can be determined in real-time by monitoring the resonant frequency of the backscattered signal due to the change of capacitance, while suture breakage leads to a change of receiving power due to the reduced electrical length (Fig. 7c-ⅱ).

Wireless data communication and remote manipulation have drawn increasing attention in smart bioadhesives, especially with the advances in the flexible electronics and chip industry. Low-power Bluetooth and Wi-Fi endow bioadhesives with real-time monitoring and on-demand treatments, which has greatly expanded their application [[22], [23], [24]]. In a simple demonstration, a smart bandage composed of a temperature sensor and drug delivery system controlled by a flexible circuit board was able to release the anti-inflammatory drug sodium salicylate using temperature feedback [24]. Since the drug was loaded on an electrochemical electrode, the timing, dose, and rate of drug release can be controlled by the magnitude and duration of the applied potential. Due to the integration of an NFC coil, and the resulting compact size, the system also has potential applications in implanted devices. In another work, more sensing modules, including both pH and uric sensors, have been integrated into the closed-loop system to provide a more comprehensive evaluation of the wound healing process (Fig. 7d-ⅰ) [22]. The smart wound dressing has a double-layer structure with a reusable flexible circuit board (the top layer) for signal processing and a disposable electrode array for sensing and drug delivery (the bottom layer). The sensing and drug delivery module was built on a polyimide substrate with a serpentine layout by laser cutting to afford stretchability and improve wear comfort and operation stability upon deformation. Data communication and powering were performed wirelessly by smartphones based on NFC designs (Fig. 7d-ⅱ). *In situ* animal studies verified the effectiveness of the smart wound dressing via faster and better wound recovery. *S. aureus* infected wounds show significantly higher temperature, pH, and uric acid concentration compared to the uninfected. After 9-days recording, the wound area with treatment was reduced to 0.032 cm^2^, which is considerably smaller than the wound without treatment (0.11 cm^2^). With a temperature sensor as the indicator, a smart bioadhesive with a double-layer structure was able to release antibiotics on demand for the treatment of infected wounds [121]. The upper layer is an integrated flexible device encapsulated by PDMS that contains a temperature sensor and UV light-emitting diodes (UV-LEDs), and the lower layer is a UV-responsive antibacterial hydrogel that can release antibiotics upon UV irradiation (Fig. 7e-ⅰ) [121]. This dressing is used for early infection diagnosis through real-time temperature monitoring and on-demand infection treatment. As shown in Fig. 7e-ⅱ, the temperature at wounds is continuously monitored by the temperature sensor and wirelessly transmitted to a smartphone by Bluetooth. The wound is diagnosed as infected when the wound temperature continuously remains higher than a preset threshold value (normally 1–2 °C higher than that of normal skin) for a given period (6 h). Then, the integrated UV-LEDs can be turned on remotely in a customized app to trigger antibiotic release *in situ* for on-demand treatment. In animal wound-infection models, full-thickness wounds were created on Bama mini pigs’ backs and then inoculated with a suspension of *S. aureus*. Afterward, the wound was treated by the UV-stimulated antibiotic release. In contrast, wounds without any treatment or treated solely by UV radiation were used as control groups. After 2 days, the density of bacteria colonies in the wound treated by the smart adhesive decreased ∼20%, while that in the control group remained unchanged. This result demonstrates that this smart bioadhesive is capable of monitoring the real-time temperature of the wound, detecting wound bacterial infection, and providing timely and effective need-based treatment.

## 6 Conclusions and future perspectives

**Fig. 8:**
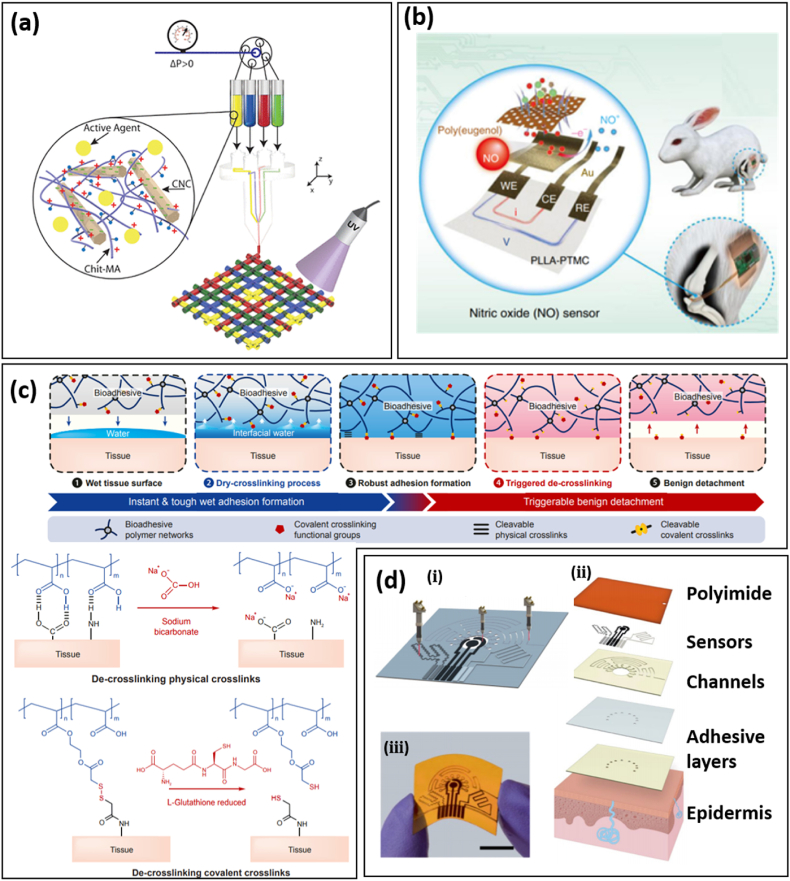
Perspectives on novel bioadhesives and biosensors. (a) 3D printing wound dressings with controllable chemical and medical distribution. Different biologically active agents, including antibacterial silver nanoparticles and vascular endothelial growth factor (VEGF), can be discretely and precisely controlled within the 3D printing technique. Particularly, with the same total amount of VEGF, the release profile of the growth factor can be slowed down by reducing the number of VEGF-loaded filaments in the meshed wound dressings. Reprinted with permission from Ref. [122]. **(b)** Schematic illustration of a biodegradable electrochemical NO sensor based on a poly(eugenol) nanolayer modified working electrode. Reprinted with permission from Ref. [19]. **(c)** A bioadhesive based on a dual-network of polyvinyl alcohol (PVA) and poly(acrylic acid) (PAA) grafted with cleavable *N*-hydroxysuccinimide(NHS) ester with triggerable detachment. The hydrogen bonds between carboxylic acid groups of PAA and tissues can be cleaved by applying sodium bicarbonate, while the covalent bonding between NHS and amine groups of tissues can be disabled by breaking the disulfide bond of NHS through applying glutathione (GSH). Reprinted with permission from Ref. [128]. **(d)** All-laser-engraved flexible devices with simultaneous sweat sampling, chemical (uric acid and tyrosine) sensing, and vital-sign (temperature and strain) monitoring. **(i)** CO_2_ lasers were employed in the fabrication of the electrochemical, temperature and strain sensor, and microfluidic channels. Exploded view **(ii)** and optical image **(iii)** of the all-laser-engraved device (A laser-engraved wearable sensor for sensitive detection of uric acid and tyrosine in sweat). Reprinted with permission from Ref. [136].

We have reviewed the latest developments of hydrogel bioadhesives inspired by natural adhesion phenomena, highlighting the importance of materials and structural engineering in the design. We make the case that a bottom-up material design methodology is necessary to optimize the performance and functionality of bioadhesives, including adhesion, cohesion, biocompatibility, antibacterial capability, and even controllable drug delivery. Traditional fabrication and application of hydrogel bioadhesives were mainly based on manually mixing and smearing on wounds, thus it is difficult to control the morphology of hydrogel bioadhesives and the spatial distribution of loaded drugs. Advanced methods (such as microfluidic generation, electrospinning, and 3D printing) have been adopted in the preparation of customized wound dressings for improved wound treatments [74,[122], [123], [124]]. With a microfluidic water-oil emulsion approach, a continuous pre-gel aqueous phase was reformatted to microgel building blocks of a microporous gel scaffold. The porous structure of the gel scaffold facilitates the infiltration of cells and promotes tissue ingrowth to balance scaffold degradation. In another work, a super-lubricated hydrogel microsphere surface modified by poly (dopamine methacrylamide-to-sulfobetaine methacrylate) (DMA-SBMA) copolymer loaded with the anti-inflammatory drug DS was proposed for osteoarthritis treatment [123]. Due to the tenacious hydration layer around the charged headgroups (-N+(CH_3_)_2_- and –SO_3_-), the DMA-SBMA modified hydrogel microsphere shows a much lower coefficient of friction than those without surface modifications, which is helpful for pain relief. It has been demonstrated that 3D printing can be used to fabricate mesh-shaped customized wound dressings with biologically active agents and antibacterial loading to address the needs of a specific patient (Fig. 8a) [122]. It is interesting to note that the drug release rate is controlled by its distribution in meshed hydrogel dressings. With the same total amount of VEGF, the meshed hydrogel with more VEGF-containing filaments has a higher release rate, possibly due to a larger surface area.

It has come to our attention that the gas environment, especially the O_2_ [112,125,126] and NO [73,112,127] concentration, is a valuable indicator of the infection and wound healing stage. Despite tremendous research on sustainable O_2_ and NO delivery or scavenging, real-time gas sensing in wounds has barely been demonstrated, possibly due to the challenge in measuring the gas in solvents. Recently, a biodegradable electrochemical sensor based on a poly(eugenol) nanolayer has demonstrated high specificity and sensitivity towards NO (Fig. 8b) [19]. It allows continuous NO monitoring in living animals for ∼7 days without side effects. It is believed that the development of bioadhesives with multiplexed gas sensing and regulation capabilities will greatly improve the efficacy of smart bioadhesives.

It is highly desirable to achieve reversible adhesion and release for wounds that require frequent medicine replacement or recycling of biosensors integrated on bioadhesives [[128], [129], [130]]. On-demand detachment has been achieved in a hydrogel bioadhesive composed of polyvinyl alcohol (PVA) and NHS grafted poly(acrylic acid) (PAA) (Fig. 8c) [128]. The hydrogen bonds between carboxylic acid groups of PAA and tissues can be cleaved by applying sodium bicarbonate, while the covalent bonding between NHS and amine groups of tissues can be disabled by breaking the disulfide bond of NHS using applied glutathione (GSH). Biological creatures, such as Echinoderms, nematodes, and parasitic flatworms, feature temporary or reversible adhesion via duo-gland organs (i.e., adhesive (viscid) gland cells and releasing (de-adhesive) gland cells) [130]. In a study on a flatworm called Macrostomum Lignano, it was found that two important proteins secreted by the gland cell (i.e., Mlig-ap1 and Mlig-ap2) are responsible for adhesion and cohesion, respectively [129]. This may spur the development of bio-inspired reversible adhesion in future bioadhesives.

Another issue in smart bioadhesives is the high fabrication cost of biosensors due to the involvement of deposition and photolithography. Reducing the fabrication cost with alternative methods, such as screen printing [131], laser cutting [22], and scribing [113,[132], [133], [134], [135]], will promote practical applications of smart wound adhesives and improve the potential for commercialization. Laser-induced graphene (LIG), featuring porous structures with a high specific surface area, can be easily fabricated into electrochemical sensors by CO_2_ laser scribing. By simple chemical modification, LIG-based electrochemical sensors have been used to monitor pH, temperature, UA, and tyrosine (Tyr) [113,132,136]. In a representative study, a sensing patch integrated with microfluidic channels was obtained entirely by laser fabrication (Fig. 8d) [136]. Using differential pulse voltammetry, UA and Tyr levels were obtained via a three-electrode setup with a LIG working electrode without specific chemical modifications and no interference between UA and Tyr sensing due to their different oxidation peaks. Considering the complex physiological conditions of wounds, decoupling the effects from different factors (e.g., temperature, strain, moisture) in dressing design is essential for precise evaluation of the healing stage.

## Declaration of interests

The authors declare that they have no known competing financial interests or personal relationships that could have appeared to influence the work reported in this paper.
